# “They still phone even though they know I'm deaf”: exploring experiences of deaf people in health services in Wales, UK

**DOI:** 10.1093/pubmed/fdae112

**Published:** 2024-06-27

**Authors:** Julia Terry, Rhian Meara, Rachel England

**Affiliations:** School of Health and Social Care, Faculty of Medicine, Health and Life Science, Swansea University, Swansea, Wales SA2 8PP, UK; Department of Human Geography, Faculty of Science and Engineering, Swansea University, Swansea, Wales SA2 8PP, UK; School of Health and Social Care, Faculty of Medicine, Health and Life Science, Swansea University, Swansea, Wales SA2 8PP, UK

**Keywords:** Deaf, hard of hearing, health professionals, Deaf awareness, patient experience communication

## Abstract

**Background:**

Deaf and hard of hearing people persistently experience barriers accessing health services, largely due to ineffective communication systems, a lack of flexible booking arrangements, and a lack of Deaf awareness training for health professional staff.

**Methods:**

Face to face focus groups were conducted with 66 Deaf and hard of hearing people in Deaf clubs across Wales, UK. Thematic analysis was undertaken.

**Results:**

Responses identified from focus groups are reported as barriers faced using health services, improvements that would make a difference, impact of accessibility of health services, and a potential Sign language badge for healthcare staff.

**Conclusions:**

Deaf people report that health professionals lack training on Deaf awareness and do not know how to communicate effectively with Deaf and hard of hearing people. Further research into Deaf awareness and training resources for health professionals are needed to establish what improves Deaf cultural competencies, and ultimately makes healthcare experiences more positive for people who are Deaf.

## Introduction

One in five adults in the UK are Deaf or hard of hearing,^1^ and the communication barriers deaf people face can compromise the trust and engagement needed at healthcare appointments. Inaccessible experiences may adversely affect early diagnosis, timely care, and adherence to required treatment.^2^ The achievement of patient-centred outcomes and full patient engagement are often problematic; and consequently, Deaf and hard of hearing people disproportionately experience persistent health inequalities both in terms of access to services and health outcomes.^3^^,^^4^ A one size fits all approach does not work in health services as this perpetuates existing inequalities.

Health professional training has been said to have a medical or deficit model focus, and still lacks information about how to effectively communicate with Deaf and hard of hearing patients.^5^ The heterogeneity of Deaf and hard of hearing people are often not fully known to health staff.^6^

Deaf with a capital D, usually refers to people who identify as, and are culturally Deaf and may be Sign language users; and deaf people with hearing loss are referred to with a lower-case d for deaf. In this paper, we use the terms Deaf, deaf, and hard of hearing.

Deaf people are twice as likely as hearing people to experience mental health problems for a range of reasons including inequality of access, limited availability of reasonable adjustments, poorer education provision, and lower rates of employment.^7^^,^^8^ Wales is very rural in places, meaning people may need to travel for healthcare. Wales is the only UK country without a Deaf mental health service, meaning Deaf and hard of hearing patients may need to travel to England for mental health inpatient provision.^9^ There are few British Sign Language (BSL) interpreters in Wales,^10^ no interpreter training programmes in Wales, which contributes to the scarcity of interpreter provision.

The first stages of the study are reported here and involved engagement with Deaf communities across Wales, UK, with data informing content for a Deaf awareness package. Our primary objective for this phase of the study was to engage Deaf and hard of hearing people and key stakeholders to clarify issues, enablers and barriers that Deaf people experience using health services. Further project objectives such as identifying specific requirements for a digital eLearning package and testing the eLearning package are reported elsewhere.^11^ Although the study focuses on a particular geographical area, the issues are far from unique, and apply to many other locations nationally and internationally.^12–14^

## Methods

Methods are reported in line with the Consolidated criteria for Reporting Qualitative research.^15^

### Sample and data collection

There are at least 20 Deaf Clubs in Wales, UK, that provide social spaces for Deaf and hard of hearing people. Each club was contacted about this project, but due to the timing of the research, not all clubs had reopened following the period of Covid-19 lockdowns in Wales, UK. Positive responses were received from seven Deaf Clubs, most clubs include a mix of Deaf BSL users and members who are hard of hearing.

The project was advertised to participating clubs using written leaflets that included a link to a video presented using BSL with English captions. This video provided further information and context about our study. Inclusion criteria noted that participants must be aged 18 years or over, must live in Wales, and must identify as Deaf or hard of hearing. We welcomed participation across genders, ages, and a diverse range of backgrounds to ensure attention not only to Deafness and its associated barriers, but also intersectional experiences of the participants. 66 Deaf people from across Wales participated in 10 focus groups. 22 participants identified as male, and 44 identified as female, with no participant drop-out. Each Deaf club visit was for approximately 90 minutes, with notes recorded across 41 pages of flip chart.

Focus groups were held between April and September 2022. Members of the research team were present at each focus group. Both the Principal Investigator and the Co-Investigator are hearing and have learnt, or are learning BSL, and the Research Assistant is Deaf and a fluent BSL user. All the research team were female, with no previous relationship with participants. Professional BSL/English interpreters were also present to ensure accessibility, clear communication and understanding. Participants were able to see the intended discussion points in advance, and the information sheet and consent forms were provided both in writing and in BSL at the start of each session.

Each focus group included a mix of Deaf BSL users and hard of hearing participants. The focus groups were not audio-recorded as most participants were using BSL as their first language. All discussion points from participants were recorded using flip charts, pens and post-it notes for each group to see, and all comments were anonymous and non-identifiable.

Each session started with a short talk about the development of the Deaf Awareness e-learning package and an opportunity for participants to ask questions. BSL/English interpreters were involved throughout. Each session ended with a debrief, and support was on hand from each Deaf Club coordinator. The discussions focused on:

Q1 - What are the biggest barriers you have faced using health services?

Q2 - What improvements would you like to see that would make a difference when you use health services?

Q3 – What has been the impact of the accessibility/inaccessibility of health services on you?

Q4 - What is your opinion of health workers wearing an "I know basic BSL" badge?

### Data management and analysis

Flipchart notes were typed, read and re-read by all authors. As focus groups progressed, researchers noted similar explanations were evident and data saturation had been reached. Themes were identified inductively with data then themed separately by two of the researchers, who then met to jointly discuss themes identified, with any discordant themes discussed until reaching consensus. Themes were then finalized and reviewed by all three authors.

### Ethical approval

The project was reviewed and approved by the School of Health and Social Care’s Research and Ethics Committee (Reference 191119c).

## Results

The findings are presented as responses to the questions in focus groups, with sub-themes. Quotes related to each theme are provided in text boxes 1–4. Detailed demographic information was not collected to maintain anonymity as Deaf communities in Wales, UK, can be small, connected groups, and demographic descriptions were not a requirement for the study. Barriers were the most commonly occurring topic with 204 comments, followed by 144 comments made about suggested improvements to health provision.

**Box 1:** Examples of participants’ comments on theme ‘Barriers faced by Deaf people using health services’

***Inaccessibility of health services:*** Medical jargon. What does it mean? Picked up the [hearing] loop, what do I need that for? Not even on. Staff have no clue how to switch it on. Most hearing aids are digital now Doctors calling by phone and having to go to surgery to find out why they called ***Difficulty booking appointments:*** Only accept phone calls. I've driven here. I can't phone. I was here first Arrived ill to make urgent appointment, can’t family ring? Could be private, I don't have to share that. I should be able to book independently ***Missing information on medical records:*** I don't know if Deaf is on my records Fed up, staff never look at records, makes it stressful unnecessarily ***Arriving and during health appointments:*** Only person left in waiting room, as people called in verbally· No LED screen downstairs, only upstairs, still calling out name downstairs so missed appointment If I take someone with me, they talk to them and not me, they look at other person Masks at cochlear centre, 'I can change it for a clear one', 'you are working in a Deaf centre should be common sense' When told ‘you can go home’, no explanation, no letter, no information, no results ***Access to BSL/English interpreters:*** No interpreters available for emergencies or out of hours Long hospital stays without interpreters (4 months+) Consent is impossible to give without interpreters. Lack of understanding of who books interpreters. Online interpreter using iPad, not great when poor Wi-Fi I fell and hurt my hip and there was no interpreter had to sit with my daughter who came with me, if interpreter at hospital no problem but using family invades privacy

**Box 2:** Examples of participants’ comments on theme ‘Improvements that would make a positive difference to Deaf people’

***Accessible information for Deaf patients:*** A welcome video in BSL More visual information especially at big hospitals Accessible information (breast cancer, peer support groups) Explain what they are going to do before asking you to remove hearing aid If giving a deaf patient an injection, it is important to gesture where it will be administered i.e. arm , bottom, etc. Someone to write notes down when a group of nurses or doctors are having a discussion about you Strong visual instructions on display when inside scanners like MRI e.g. Don’t move, 2 minutes left, etc. ***Equipping staff:*** Staff to have basic sign language, it’s about reducing stress Paramedics need to know sign language and deaf awareness to explain things like why you cannot have a drink or some food, and why going into hospital and could be hours before the doctor tends to you and explains everything Develop something on system to say Deaf Maybe need more Deaf awareness training not just once through job ***Systems that empower Deaf people:*** Why can't we book interpreters directly? Visual relay system, could find information, all should have contract Visual relay service Longer appointments face to face Email, text or text relay as options to book appointments independently Hospitals are the ones that need cultural changes ***Improvements needed relating to interpreter provision:*** Each hospital with a qualified experienced interpreter Have more out of hours interpreters on the books Someone available 24/7 with basic BSL skills Budget for interpreters who always work in hospital settings, cover shift patterns, had this cover in past

**Box 3:** Examples of participants’ comments on theme ‘impact of accessible/inaccessible health services on Deaf people’

**No sign of service improvements** *When I made a complaint 6 weeks ago about no interpreter in an emergency appointment there has still been no improvement and I had to go in an ambulance due to a mental health breakdown* *Been saying this for years, why still happening?* **Coping and realisation** *One Deaf man happy without interpreter; then started booking them as he didn’t realise himself how much info he had missed out on* *Put in complaint* *Early morning, often no interpreter, emergency, happened 5 times, have coped without.* **Use of resources** *Had 5 interpreters turn up, waste of time and money* *When you could book [GP appointments] online it was better, I could pick which doctor was best to lipread*

**Box 4:** Examples of participants’ comments on theme ‘Potential Sign language badge for healthcare staff’

We asked participants what they thought of the idea that healthcare staff with basic Sign Language knowledge might wear a badge, so Deaf people who use Sign Language might identify these staff. **Potential advantages of a basic BSL badge:** *Might prefer to talk to badge wearer* *Train receptionists in BSL* *You’d know if someone could sign* *Good idea to say you are Deaf aware* *Just use for simple translation to help calm person and not to be used as an interpreter* *As part of training nurses and doctors* **Potential disadvantages of a basic BSL badge:** *Just because someone has basic BSL does not mean they have a good deaf aware attitude* *Good idea for when booking or discussing appointments but not regarding health information*

### Barriers faced by Deaf people using health care settings

All focus group participants explained in-depth about the barriers faced by Deaf people using health care services in Wales. Although many discussion points focused on access to GP doctors and hospitals, discussion was by no means limited to these settings and included access to wider healthcare providers such as Dentists, Opticians, Pharmacies and Antenatal Services. Participants discussed personal current and past experiences. Several sub-themes were identified in this category, and these are discussed below with quote examples in Box 1.

#### - Inaccessibility of health services

Several participants noted limited communication at healthcare settings (e.g. no interpreters) meant patients were left without clear instructions or in some cases misunderstood information. Participants were aware there could be negative consequences for their health if, for example, misunderstandings related to appropriate dosages of medicines or fasting before operations.

### - Difficulty booking appointments

A regular theme in focus groups was difficulties making appointments. Most doctors’ surgeries in the UK require patients to telephone to make an appointment. Many surgeries expect patients to have a triage phone call before an appointment can be confirmed. Some surgeries do now have online appointment booking forms, but typically this is followed up by a phone call with a doctor, thus limiting access to Deaf patients. Equally reception staff were suggesting family members/friends could phone on their behalf to make appointments. But this impacts heavily on patient confidentiality, and assumes patients have strong support networks. It is widely recognised that involving family members to assist or to interpret is not accepted in the making of or involvement in health appointments.

Participants noted appointments for Deaf patients typically ran late and they felt as though they were treated differently to hearing peers. For example, Deaf people reported seeing they were often the last patient left in the waiting room, had missed their turn and perceived hearing patients to be having an easier experience than they were.

#### - Missing information on medical records

Participant noted that they were unsure if Deafness was recorded on their medical records. They felt that this was the only explanation for the continued lack of understanding or support needed by patients. Others noted information was on their medical records but was consistently overlooked, meaning appointments were impacted by a lack of preparedness by medical staff.

#### - Access to BSL/English interpreters

Access to qualified BSL/English interpreters was a common discussion point across all 10 focus groups, with frequent misunderstandings between healthcare settings and patients as to who was responsible for booking interpreters. Booking interpreters is not a patient responsibility. Participants were aware that whether or not they needed an interpreter should be information on their health records, but often they reported this detail was absent.

Additionally, there are complications linked to accessing interpreters at short notice, as many UK GP surgeries only provide same-day appointments and do not allow you to book appointments in advance. Many healthcare providers did not know about alternative/remote video communication strategies such as SignLive^16^ or SignVideo.^17^

### Improvements that would make a positive difference to Deaf people

Participants had helpful suggestions about how health services could be improved for Deaf and hard of hearing people.

#### - Accessible information for Deaf patients

Ideas flowed particularly around welcomes as patients arrive, instructions and information, with clearer visuals, which would reduce anxiety for Deaf and hard of hearing patients. Participants were aware appointments go more smoothly if they are able to respond to instructions clearly, e.g. during procedures that required lying still. Welcome videos in BSL with captions were also suggested.

#### - Equipping staff

Commonly, participants felt health staff should be better equipped with Deaf awareness training and basic BSL signs, showing Deaf patients they were expected and welcome in the space. Having staff who know how to communicate about basic needs like where the toilet is or whether patients are allowed to eat and drink would be helpful, especially if it may be hours before seeing a doctor/nurse who might explain.

#### - Systems that empower Deaf people

Suggestions were made about Deaf people being empowered to book BSL/English interpreters, particularly as participants were aware few staff knew how to do this efficiently. Participants acknowledged this was not their responsibility but reported they would feel more confident if they had greater involvement in the process and knew what was happening with interpreter provision for each health appointment.

Increase in time-length of appointments was suggested as participants knew from experience, they frequently left appointments without clear explanations of instructions about their care and treatment. Explanations centred on improvements being practical, and Deaf and hard of hearing patients not feeling they were a burden, but were equally provided for.

#### - Improvements needed relating to interpreter provision:

Participants reported the benefits of having access to 24/7 BSL/English interpreters, particularly in areas like unscheduled care, and were aware that some hospitals had delivered these services successfully in the past.

### Impact of accessibility / inaccessibility of health services

Participants explained the impact on themselves were health services to be more accessible, as well as consequences they had experienced due to difficulties. Negative impacts on people’s mental health were commonly stated.

#### - No sign of service improvement

Participants who had complained about their care previously noticed they did not experience improvements or see any changes, and felt they were battling services.

#### - Coping and realisation

Participants who felt they had put up with the status quo, and then asked for different provision stated that they were amazed at how much they had missed out on previously. Frequently, participants explained they accepted services would be inaccessible and were used to coping with a different level of service to hearing people.

#### - Use of resources

Participants highlighted they were aware due to service inefficiencies and red tape, sometimes no interpreters were booked and sometimes in error several were booked when not requested. Participants gave these examples as a poor use of resources.

### Potential sign language logo for healthcare staff

Staff working at healthcare settings in Wales are expected to highlight their Welsh language abilities on their uniforms or scrubs. During focus groups, we introduced participants to the logo worn by Welsh Language Speakers and learners, typically worn as badges to note ability to speak Welsh. Participants were asked whether they thought introducing similar logos relating to BSL would be valuable in healthcare settings, with potentially a logo for fluent BSL users and another for those learning. Groups noted concerns about Deaf people themselves wearing such obvious logos as it would draw attention to potentially vulnerable individuals highlighting their deafness. We clarified logos would be for those working in healthcare settings highlighting to Deaf patients which staff were able to communicate using BSL.

Comments were mixed (see Box 4), with concern that the use of BSL logos might breed false confidence, for example the actual abilities of staff who sign may be over-estimated by badge-wearing individuals, or potentially that Deaf BSL users could have high expectations and might over-estimate BSL abilities of staff. There was a strong feeling that while this would be a welcome addition in principle, there was a fear underqualified staff would be urged to act as unofficial interpreters for Deaf patients. It was also highlighted that Deaf communities would need to be made fully aware of the meaning of the new logos, and ideally consulted on their design and implementation. Overall, there was general support in principle for the idea of a badge, and particularly that health professionals needed to have some level of proficiency in BSL.

## Discussion

### Main findings of this study

This study highlights ongoing challenges Deaf people experience as they attempt to engage with health services for appointments, medical tests and treatments. Although there were occasional positive experiences in health services reported by focus group participants, in the main Deaf people reported a catalogue of disadvantages they have experienced over many years and stressed they had seen little signs of improvement. Participants acknowledged during the main lockdown periods of Covid-19 they experienced increased difficulties with health service engagement. In the main Deaf patients find health services largely inaccessible with problems occurring frequently relating to bookings, arriving and checking in at appointments and during consultations, medical tests, treatment and hospital inpatient stays. Across all focus groups in all locations similar themes and comments arose, suggesting commonalities around challenges and poor access. Whilst in all groups some participants did mention local services where they had had positive experiences, similar negative experiences arose time and again, suggesting data saturation.^18^

Due to limited access, resource and provision, Deaf participants relied heavily on family and friends for health information, and sometimes for booking appointments and BSL interpreting, even though they acknowledged this was not appropriate in terms of privacy, appropriateness, and sometimes inaccurate health information.

### What is known on this topic

Previous research suggests Deaf people experience poorer physical and mental health and have poorer health literacy than the general population.^19–22^ Deaf community members are at increased risk of preventable ill-health, with chronic health conditions likely underdiagnosed and undertreated.^23^ Deaf populations have reported not being satisfied with medical care.^24^ One healthcare service specifically identified has been emergency care with Deaf communities reporting substantially poorer access compared with the general population.^25^^,^^26^ Further examples of a lack of health service provision also relate to mental health care with many countries without a specific Deaf mental health service.^8^^,^^9^

Sign language users in a range of countries reported their distinct language, sensory and Deaf cultural profile are not recognised by health professional staff.^27^^,^^28^ This lack of recognition can result in Deaf individuals’ values and citizenship rights not being upheld.

Subsequent consequences can result in Deaf people avoiding seeking care, having limited access to health information and healthcare providers, and for Deaf women during pregnancy, birth and postnatal periods, this can mean having longer hospital stays.^29^ Attempts at improvements by trialling specific Deaf health clinics, and increasing the number of providers who understand the social and community context the patient comes from have had some success.^30^

Evidence also indicates a need for increased inclusion of Deaf awareness programs, including how to communicate with Deaf people in health professional curricula, which would ensure increased Deaf cultural competencies.^31–33^ In achieving these aims, consultation with Deaf people is essential as Deaf and hard of hearing people teach hearing staff that connection is not just about sound, but that communication is visual.^34^

### What this study adds

Whilst many of the views expressed by participants in this study from Deaf clubs in Wales, UK, reinforce findings of previous studies, our experience indicates the topic is of great interest and importance to Deaf communities.

Participants stated a need for increased BSL interpreters to be trained and available. Whilst this change is wanted by Deaf BSL users, there are still no training programmes for BSL interpreters in Wales, so numbers are likely to remain at a low level, with currently only around 30 BSL/English interpreters registered. There were many participants who conveyed a need for changes to the ways that BSL interpreters are booked and confirmed, with Deaf people wanting increased autonomy, choice, communication, and decision-making in the process, as the current system is reportedly to be ineffective, with few successful stories expressed; mostly due to frustrations and booking processes that are unclear to health staff and Deaf people alike, although no clear solution was proposed by participants.

Many participants expressed feelings of fear, distress and avoidance of engaging in health services often due to poor past experiences, reporting they would likely feel isolated and have limited access to information and expected a less satisfactory experience than hearing patients. There was generally a preference for a badge for health professional staff indicating if they have a basic knowledge of BSL as participants suggested this would at least indicate a person in a health setting that they might approach for basic information.

These findings have implications for health professional education providers and health service providers who have ignored complaints raised by Deaf patients. The Equality Act 2010 was mentioned by several participants in this study, as well as awareness that despite promises for positive change, Deaf people in Wales, UK have so far seen little improvement in their health service experiences. According to participants in this study, improvements are needed in booking systems and accessibility of services. There is also a need for an increase of BSL/English interpreters, particularly in Wales, and efficient systems for health staff to book interpreters, with Deaf people involved in processes, so they are fully informed and confident about arrangements. Participants also want to see improvements to data coding and categories, so they are confident their language and communication needs are included and visible on all health records, so each worker immediately knows their requirements. A further improvement would be that all health staff have working knowledge of BSL and can engage in basic communication and greetings.

### Limitations

This study cannot claim to be representative of all Deaf people in Wales UK. However, Deaf clubs across different parts of Wales, UK, were represented (including North, South, West and South-East Wales) and were held both during day and evening times to offer choice for those who may have been working, caring or retired. We also sought a range of participant demographics in terms of age, Deaf Sign language users and those who preferred to use speech, Cochlear implant and hearing aid users as well as those who do not use assistive technology. The research team acknowledge that there may be many other Deaf and hard of hearing people in Wales who use health services and do not connect with Deaf clubs.

## Conclusion

Deaf and hard of hearing people frequently experience challenges and barriers when they attempt to engage with and attend healthcare services. These challenges and barriers often relate to limited accessibility booking appointments, to limited health information and to health professionals having little Deaf awareness training. It is vital that healthcare providers are properly trained and prepared to meet the needs of Deaf people which primarily starts with effective communication.

Further research is needed to examine which methods and training resources are most effective in improving Deaf cultural competencies, knowledge and skills of health professionals and how these in turn may significantly improve Deaf people’s healthcare experiences for the better.

## Supplementary Material

Coreq_checklist_They_still_phone_me_even_though_they_know_bbae172

## Data Availability

The data in this article will be shared on reasonable request to the corresponding author.

## Notes

**J. Terry,** Dr

**R.H. Meara,** Dr

**R. England,** Research Assistant
